# Effects of a group-based music imagery program on promoting coping resources among undergraduate students: a pilot randomized controlled trial

**DOI:** 10.3389/fpsyg.2023.1257863

**Published:** 2023-12-01

**Authors:** Winnie Lai Sheung Cheng, Lokki Lok-Ki Wong

**Affiliations:** ^1^School of Health Sciences, Caritas Institute of Higher Education, Tseung Kwan O New Town, Hong Kong SAR, China; ^2^School of Nursing and Health Studies, Hong Kong Metropolitan University, Hong Kong, Hong Kong SAR, China

**Keywords:** self-compassion, stress mindset, music therapy, sense of coherence, stress management, randomized controlled trial, pilot study

## Abstract

**Background:**

Music is well-known for its stress-reducing effects. Little is known about the potential effect of music interventions in fostering internal coping resources for stress management among undergraduate students in Hong Kong.

**Objectives:**

This pilot study aimed to examine the efficacy of the Group-based Focus Music Imagery Program (GFMI) in promoting a stress-is-enhancing mindset, sense of coherence, and self-compassion among undergraduate students.

**Methods:**

We used a two-arm parallel randomized controlled trial (RCT). The experimental arm received 6 weeks of GFMI with measures taken at two time points after completing baseline assessments (Weeks 6, 10). The control arm received 6 weeks of an active control program and completed the outcome measures at time points similar to the GFMI group. Data were collected using the Chinese versions of the Sense of Coherence Scale (C-SOC-13), the Self-Compassion Scale-Short Form (C-SCS-SF), the Stress Mindset Measure (C-SMM), the Perceived Stress Scale (C-PSS-10), and the Generalized Anxiety Disorder scale (C-GAD-7).

**Results:**

Sixty-four participants were randomly assigned to either the experimental group (*n* = 32) or the control group (*n* = 32) between July 2021 and September 2022. The experimental group exhibited a retention rate of 71.9% at T1 (23 out of 32 participants), which slightly decreased to 65.6% (21 out of 32) at T2. The control group displayed a retention rate of 75% (24 out of 32) at T1, which dropped to 43.8% (14 out of 32) at T2. The GEE analyses showed insignificant differences between groups at any time point in C-SOC-13, C-SCS-SF, and C-SMM. Instead, the control group had a higher reduction in stress scores (C-PSS-10) at T1, and anxiety (C-GAD-7) at T2 than the experimental group.

**Conclusion:**

The pilot trial provided valuable information in examining the feasibility of the trial design and intervention. Future studies with larger samples are needed to validate if GFMI can reliably promote coping resources to manage stress and anxiety in undergraduate students.

**Trial registration number:**

https://www.researchregistry.com/, researchregistry8209.

## 1 Introduction

Undergraduate students have been well-known for their high levels of stress from multiple sources, including academic stressors and stressors associated with changes in their physical, including sexual, sense of self. They may also face new and increased roles and responsibilities within their families and society, as well as the challenges of transitioning to independent living and adulthood (Karyotaki et al., 2020). Previous studies reported that high level of stress undermines psychological well-being (Wang et al., 2021) and is associated with mental disorders (Karyotaki et al., 2020). People with unhealthy coping strategies or mechanisms typically report higher levels of stress (Böke et al., 2019). Effective coping is thus important to managing stressful events. According to Lazarus and Folkman (1984), coping resources, both personal and social resources are important in stress management as they influence the strategies available to individuals. Better personal resources help reduce distress reactions when encountering stressors, and the presence of such abilities enables individuals to stay healthy (Sagy and Braun-Lewensohn, 2009).

In the past two decades, interest in using music-based interventions with undergraduate students have grown. Music-based interventions can encompass a diverse range of approaches, including both active music therapy techniques that involve direct interaction with a trained music therapist to influence clinical outcomes, as well as passive music listening activities (Raglio and Oasi, 2015). Fiore (2018) reported that using receptive music could reduce students’ stress and anxiety levels. Other researchers also reported positive results in reducing stress when music therapy combined with muscle relaxation (Gallego-Gómez et al., 2020) and aromatherapy (Son et al., 2019). Music intervention could be a strategy to reduce stress. However, the mechanism of how music intervention works on personal coping resources has not been ascertained. The purpose of this pilot study is to determine whether a group-based music relaxation program has a significant impact on coping resources among undergraduate students.

The present study investigates three internal coping resources: stress mindset, self-compassion, and sense of coherence. A stress mindset is a belief that stress can have strengthened, rather than debilitating, properties (Crum et al., 2017). It is regarded as a positive internal resource. Individuals who believe that stress can enhance stress-related outcomes cope better with stress (Crum et al., 2013; Kilby and Sherman, 2016). Self-compassion is a psychological construct that involves treating oneself with kindness, care, and understanding in the face of personal difficulties, mistakes, and failures (Neff, 2003). People who practice self-compassion are kind to themselves and avoid being overly self-critical, even when facing negative experiences (Neff, 2003). Sense of coherence is based on the belief that life events are predictable and manageable, and that one’s life has meaning and purpose (Antonovsky, 1987). This belief can strengthen the sense that one has adequate resources to manage the demands of life events (Antonovsky, 1987, 1993). Individuals with a strong sense of coherence are better equipped to understand and navigate life’s challenges, and cope with stress and tension (Nielsen et al., 2008).

Listening to music has a significant impact on emotional regulation by influencing various functions within the brain, including the motor/autonomic, emotional, and prefrontal areas (Saarikallio, 2011; Osborne, 2012). Empirical studies have consistently demonstrated the positive effects of music on emotional regulation (de la Torre-Luque et al., 2017). Therefore, music may help promote positive cognitive thinking, thus engendering internal resources in coping. However, it is important to acknowledge that music interventions encompass a wide range of approaches and techniques. It is possible that certain strategies used for musical regulation may have maladaptive effects in specific cases (Carlson et al., 2015). A systematic review has indicated that music therapy integrated with guided imagery was able to enhance well-being, quality of life, mood states, and sense of coherence in various populations, with most studies conducted on cancer patients and fewer studies on healthy adults (Jerling and Heyns, 2020). In addition, the modalities of interventions vary, with various adaptations and modifications of guided music imagery, including group and individual sessions (Jerling and Heyns, 2020). Hence, additional research is warranted to examine the effectiveness of combining music listening with guided visualization techniques in enhancing internal coping resources for stress management among healthy individuals.

## 2 Conceptual framework

The proposed study is grounded in the theory of Stress, Appraisal, and Coping (Lazarus and Folkman, 1984) and the framework of interpersonal neurobiology (Siegel, 1999). According to the theory of Stress, Appraisal, and Coping, the concept of stress is related to transaction between the individual and the environment (Lazarus and Folkman, 1984). Stress arises when an individual perceives that they are powerless to cope with the demands placed on them. Coping is a constantly evolving cognitive and behavioral effort to manage situations appraised as stressful (Lazarus and Folkman, 1984). Type of coping strategy is influenced by the evaluation of options for coping, referred to as ‘secondary appraisal’ (Lazarus and Folkman, 1984). Secondary appraisal involves evaluation of the personal resources that can be done to manage the situations. The cognitive appraisal of one’s resources including both internal and external environment resources, which is important for that individual to determine the coping strategies. Since the sense of coherence is a personal resource that enables coping with adverse situations (Antonovsky, 1979, 1990), individuals with strong sense of personal resources will have a better sense of coping ability (Lazarus and Folkman, 1984).

The principle of interpersonal neurobiology posits that there is a link between mind, brain and interpersonal experience. The function and structure of the mind and brain are shaped by “the interface of neurophysiological processes and interpersonal relationships” (Siegel, 1999, p. 21). Grounded in the theory of cognitive neuroscience, our understanding of the mind and subjective experiences has evolved to incorporate modern concepts such as functional connectivity and connectomics. Functional connectivity, a key concept in cognitive neuroscience, pertains to the patterns of neural activity and communication between different regions of the brain (Bullmore and Sporns, 2009). It is the dynamic interactions and connectivity between brain regions that contribute to the creation of the mind and subjective experiences.

Recent research in connectomics has provided valuable insights into the intricate workings of the brain, revealing the complex neural activity and communication patterns that underlie our mental representations and experiences. Neural networks, formed by the functional connectivity between different brain regions, play a crucial role in processing and integration of various types of information, including emotional, bodily, and social information (Bressler and Menon, 2010). The middle prefrontal region, which encompasses the anterior cingulate, orbitofrontal, medial, and ventral regions of the prefrontal cortex, is involved in the integration of emotional and social information (Siegel, 2007, 2020). The interactions between this region, the limbic region, and the cortex contribute to the development of an integrated brain and a coherent mind, fostering growth in relationships with oneself and others (Siegel, 2020).

Listening to music in a relaxed state and imagining positive mental images can create new neural pathways and connections in the brain (Moore, 2013), potentially enhancing personal coping resources and leading to better stress management and a greater sense of well-being.

**Figure 1:**
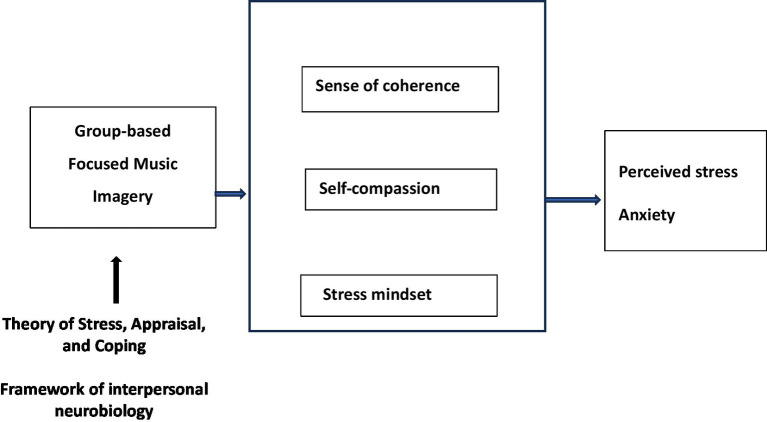
Conceptual framework of the study.

Hence, we hypothesise that a music intervention program, specifically the Group-based Focus Music Imagery program (GFMI), could provide individuals with a way to create mental images, which may enhance personal coping resources such as a positive stress mindset, self-compassion, and a sense of coherence. The GFMI program, which combines music listening with imagery techniques, is designed to mitigate potential maladaptive effects while maximizing the positive outcomes of music therapy. It is delivered by a trained music therapist who provides receptive music therapy. The GFMI program creates a safe and non-threatening environment for individuals to express and explore their emotions. With the guidance of the music therapist, the images that arise during music listening are processed in a way that allows participants to develop an understanding of them as metaphors applicable to their present situation or concerns. This process enables participants to communicate and process complex emotions, promoting emotional validation. Emotional validation is a critical step in fostering positive cognitive thinking and internal coping resources. Furthermore, the GFMI program involves a therapeutic relationship between the music therapist and the individual receiving the intervention. This relationship provides a supportive and empathetic environment where individuals can explore their emotions. The presence of a trained professional helps individuals navigate any potential maladaptive effects, ensuring that the therapeutic process remains beneficial and conducive to positive cognitive thinking. By enhancing self-awareness and emotional regulation abilities, the GFMI program empowers individuals to cultivate positive cognitive thinking and develop adaptive coping strategies. The conceptual framework of the study is depicted in Figure 1.

This paper presents the preliminary findings of a pilot study that investigates the following research questions:

1. Will GFMI enhance internal coping resources, specifically by instilling a “stress-is-enhancing” mindset, increasing self-compassion, and promoting a sense of coherence, in undergraduate students?
2. Will GFMI reduce perceived stress and anxiety in undergraduate students?

## 3 Methods

### 3.1 Study design

**Figure 2:**
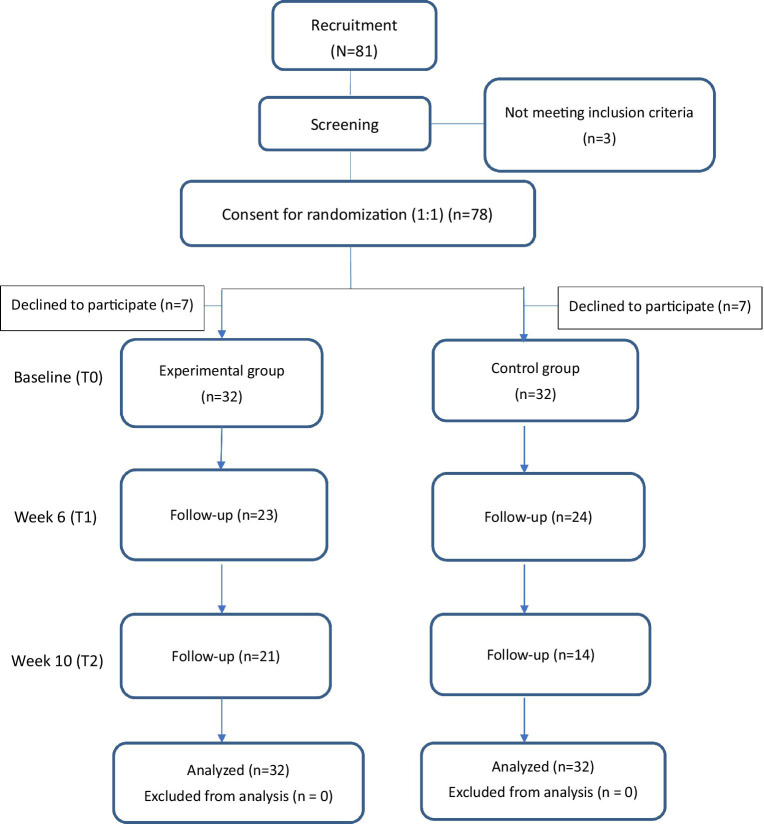
Study flow.

We used a two-arm parallel randomized controlled trial (RCT) to evaluate the efficacy of the GFMI. The experimental arm received 6 weeks of GFMI with measures taken at two time points after completing baseline assessments (Weeks 6, 10). The control arm received 6 weeks of an active control program and complete the outcome measures at the time points similar to the GFMI group. Figure 2 shows the study flow. The study was single-blinded to the participants.

### 3.2 Participants

To assess the sample size, we used G*Power software (version 3.1.9.2; Franz Foul, University Kiel, Germany). We calculated the optimal sample for the statistical tests: a repeated-measures analysis (between factors), medium (0.25) effect size, and three-time number of measurements. For the testing of two groups, the total sample size required for a statistical power of 80% is 86 participants. As for our pilot study, the suggested sample size should be 50% of the total main trial size according to Wittes and Brittain (1990). Therefore, 43 participants would be needed in the present study. We considered an initial loss of 30% of participants during follow-up; thus, 64 eligible patients were invited to participate in the study, of whom 32 were assigned to either the experimental group or the control group.

The participants were recruited from the undergraduate students of a higher education institution in Hong Kong, SAR, China, those who (1) had a history of diagnosed psychiatric disease and/or current acute mental problem, (2) had dislike of music, and (3) had previous experience with music therapy that involved mental imaging were excluded. Of the 81 invited students, an informed consent form was obtained from 78 (96.3%) students. Fourteen students declined from participating in the study before the research study started. In total, 64 students (82.1%) were able to participate in the experimental group (EG; *n* = 32) and control group (CG; *n* = 32).

### 3.3 Setting and participants

#### 3.3.1 Procedures

Participants were allocated to either the experimental group or the control group using simple randomization by computer-based two-digit random number with Excel 2007 (Microsoft, Redmond, WA, USA) after providing their written informed consent to participate in the study. Concealment of allocation was implemented to ensure that the assignment of participants to different groups was kept undisclosed until after they met all the inclusion criteria and were offered participation in the study.

### 3.4 Study intervention

#### 3.4.1 Experimental group

Internal coping resources are important because they enable individuals to handle stress in a more effective manner. Having strong internal coping resources helps individuals to better manage stress and to maintain a sense of control and well-being in the face of adversity. GFMI is a program that combines music listening with imagery techniques. Participants engaged in GFMI with the guidance and support of a music therapist. This approach allowed participants to explore and understand the images that emerged during music listening sessions. The GFMI was delivered to the experimental group. A weekly session of 120-min GFMI for 6 weeks was delivered to a group of a maximum of 10 participants by a certified music therapist with training in GFMI. Each session of GFMI comprises: (i) warm-up, (ii) Prelude, (iii) Induction & Music Listening, (iv) Postlude, and (v) Closing.

The session began with a warm-up activity aimed at helping group members get to know one another and feel more comfortable sharing in the group. In the Prelude phase of the study, participants were encouraged to identify and express their common interests, which helped to create a shared focus of feelings and establish a sense of community within the group.

The Induction & Music Listening phase began with participants entering a state of relaxation through deep breathing, followed by a brief music-listening interval (1–2 min) with their eyes closed. During this time, the music therapist used the shared focus identified earlier as a talking point and guided participants in developing mental images related to the topic. After the talk-over induction, while the music continued to play, participants were instructed to open their eyes and draw whatever image(s) emerged. The drawing activity usually lasted for 15 min, and the music ceased when it was completed.

The Postlude phase aimed to provide debriefing among the group. Participants were asked to write on their drawings a title related to the image (T), a theme related to the image (T), an affective word associated with the image (A), and a question they wanted to ask themselves (Q). The music therapist then facilitated group interaction as each participant described their images and shared the content of their TTAQ. Participants were encouraged to support each other in developing an understanding of the meaning of the images as metaphors that were applicable to their present situation.

The session ended with a closing ritual, such as a gesture or movement, to create connections among the participants.

##### 3.4.1.1 Music selection

**Figure 3:**
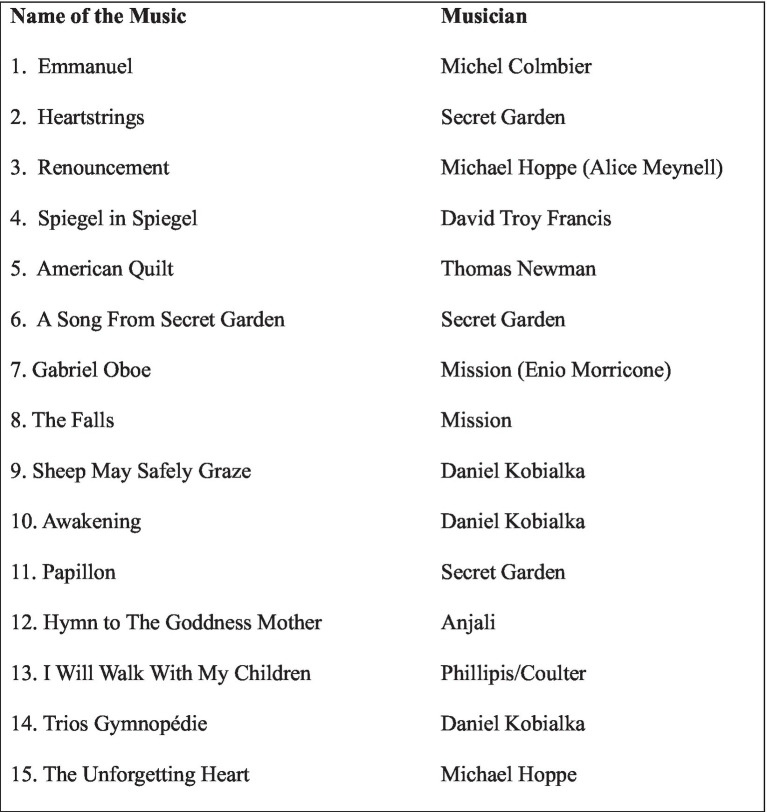
List of musical pieces.

We prepared a selection of tranquil, simple, non-vocal, non-classical musical pieces for music listening during the study in consultation with a music therapist trained in Focus Music Imagery. Examples of non-classical musical pieces included Emmanuel, Heartstrings, and Gabriel Oboe (Figure 3). The specific pieces of music chosen for the group sessions were agreed upon by the participants.

Non-classical music was chosen because it often has a more universal appeal and can be more accessible to a wider range of people. It typically has a more repetitive structure, which can be more conducive to inducing a trance-like state of relaxation and concentration (Bruscia, 2002). Additionally, non-classical music can evoke a range of emotional responses and associations, provide a variety of images, and allow individuals to explore their unique experiences and emotions in a safe and supportive environment (Dimiceli-Mitran, 2016). These qualities were especially conducive to inducing a trance-like state of relaxation and concentration, as the GFMI aimed to guide the listener into a state of relaxation and inner exploration.

#### 3.4.2 Control group

The control group participants (maximum 10 people) attended a session every week for 6 weeks. The control condition lasted for about 120 min and was delivered by two registered nurses. It comprised three activities: (i) breathing and stretching exercises, (ii) guided imagery that involves visualizing peaceful settings like a beautiful beach, and mental body scanning, and (iii) still-life drawing with pastels as music plays in the background. An everyday object such as a lamp, vase of flowers, or houseplant will be provided for drawing. The participants were instructed to draw the object without worrying whether the quality of the drawing or technical skill displayed. After completion of the drawing, participants were encouraged to share their experiences. The musical pieces were the same as those used for the GFMI group.

### 3.5 Treatment fidelity

To ensure treatment fidelity, the research team conducted regular meetings to ensure the study protocol was executed as intended. Detailed guidelines and procedures were provided to interventionists in both the experimental and control groups to promote consistency and minimize variations. Checklists were employed to verify adherence to the study protocol in both groups.

In the experimental group, the implementation of the study intervention, specifically the GFMI program, was supervised by an experienced consultant. This supervisor played a crucial role in upholding the quality of the intervention. To monitor the intervention’s integrity, group sessions in the experimental group were audio-recorded with participants’ consent. Subsequently, the recordings were transcribed and presented to the consultant for thorough quality monitoring.

### 3.6 Outcome measures

**Figure 4:**
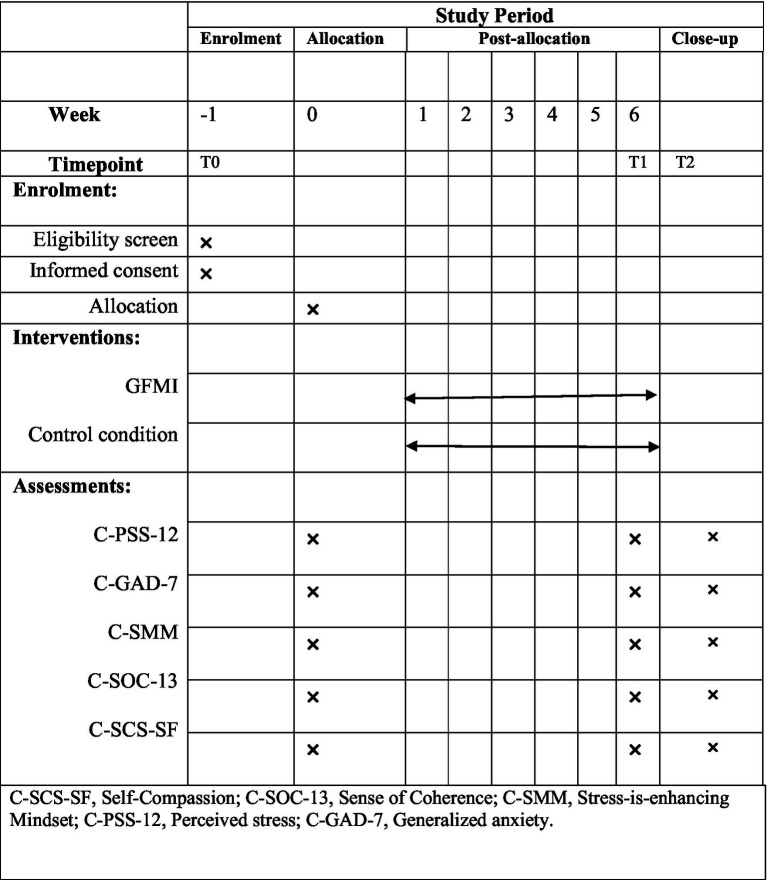
Outcomes and time points of data collection.

The outcomes were measured using the Chinese versions of Stress Mindset Measure (C-SMM), the Sense of Coherence Scale (C-SOC-13), the Self-Compassion Scale-Short Form (C-SCS-SF), the Perceived Stress Scale (C-PSS-10), and the Generalized Anxiety Disorder Scale (C-GAD-7). Data were collected before the study began (Baseline, T0), when the program ended (Week 6, T1); and 4 weeks after the program (Week 10, T2). In addition, a structured questionnaire was used to collect demographic information, including gender, age, marital status, religion, living conditions, and coping behaviors. The Chinese version of the Brief Cope Inventory (COPE) was used to measure coping behaviors (Yuan et al., 2017). Figure 4 summarizes the outcomes and time points of data collection.

#### 3.6.1 Sense of coherence

The Sense of Coherence Scale-13 (C-SOC-13) consists of 13 items across three domains: comprehensibility, manageability, and meaningfulness (Antonovsky, 1987, 1993). All items were rated on a 7-point semantic scale with two anchoring phrases, ranging from 1 (very poor) to 7 (very strong). The total score is obtained by summing the scores of all items after reversing the score of the negative items. A higher mean score indicates a higher level of sense of coherence. The original SOC-13 Scale has been shown to have high internal consistency (α = 0.74–0.91; Antonovsky, 1993) with good factor validity, predictive validity, and divergent validity, and good internal consistency with Cronbach’s alpha coefficient of 0.824 (Ding et al., 2012). The Chinese version has been widely used in different populations (Ying et al., 2007; He et al., 2012). In this study, the reliability coefficient Cronbach’s α values were 0.83, 0.88, and 0.85 across the three time points, respectively.

#### 3.6.2 Self-compassion

The SCS-SF consists of 12 questions across six domains: self-kindness, self-judgment, common humanity, isolation, mindfulness, and over-identification (Neff, 2003). All questions were rated on a 5-point scale, ranging from 1 (almost never) to 5 (almost always). The total score is obtained by summing the scores of all items after reversing the score of the negative items. A higher mean score indicates greater self-compassion. The SCS-SF has acceptable psychometric properties when used to test Chinese college students. The internal consistency was Cronbach’s alpha coefficient of 0.84, and validity was confirmed with exploratory and confirmatory factor analyses (Chen et al., 2011). It has been widely adopted in different populations (Yang, 2016; Finlay-Jones et al., 2018). In this study, the reliability coefficient Cronbach’s α values were 0.83, 0.88, and 0.85 across the three time points, respectively.

#### 3.6.3 Stress-is-enhancing mindset

The C-SMM consists of eight items that assess an individual’s perception of stress as either empowering or debilitating in response to stressful situations (Crum et al., 2013). All items were rated on a 5-point scale, ranging from 0 (Strongly Disagree) to 4 (Strongly Agree). A higher mean score indicates a greater stress-is-enhancing mindset. The C-SMM has been found to have a good fit with a single-factor structure with the original structure (*χ*^2^/df = 2.23, RMSEA = 0.047, CFI = 0.966, TLI = 0.932; Jiang et al., 2019). The scale has been widely used in college student populations (e.g., Kilby and Sherman, 2016; Jiang et al., 2019). In this study, the reliability coefficient Cronbach’s α values were 0.83, 0.88, and 0.85 across the three time points, respectively.

#### 3.6.4 Perceived stress

The C-PSS-10 consists of 10 items that assess an individual’s feelings and thoughts during the last month and indicate their perception of stress (Cohen et al., 1983). All items were rated on a 5-point scale, ranging from 0 (never) to 5 (very often). The total score is obtained by summing the scores of all items. A higher total score indicates greater perceived stress. The C-PSS-10 has been confirmed with Exploratory Factor Analysis, accounting for 62.41% of the variance (Wang et al., 2021). It has good construct validity of 0.69 and 0.72 (Remor, 2006; Maroufizadeh et al., 2014). The scale has been widely used in diverse populations (e.g., Liu et al., 2007; Lau and Yin, 2011; Lu et al., 2017). In this study, the reliability coefficient Cronbach’s α values were 0.83, 0.88, and 0.85 across the three time points, respectively.

#### 3.6.5 Anxiety

The C-GAD-7 consists of seven items that assess the extent to which an individual has been bothered by feelings of anxiety, such as nervousness, worry, and restlessness, during the previous 2 weeks (Spitzer et al., 2006). All items were rated on a 4-point scale, ranging from 0 (not at all) to 3 (nearly every day). The scores of all items are summed to provide a total score; a higher score indicates a higher degree of anxiety. The C-GAD-7 has been found to show good reliability and validity; the Cronbach’s alpha coefficient was 0.898, and the test–retest reliability was 0.856 (He et al., 2012). It has been widely used in diverse Chinese populations (e.g., Cao et al., 2020; Huang and Zhao, 2020). In this study, the reliability coefficient Cronbach’s α values were 0.83, 0.88, and 0.85 across the three time points, respectively.

### 3.7 Statistical analysis

For continuous variables, means and standard deviations were used to present the data, while frequencies and percentages were used to present the categorical variables. Generalized Estimating Equations (GEE) models were used to assess the effects between the experimental and control groups over time, while controlling for baseline variables that were significantly different between the two groups (religion, perceived stress, anxiety). The data were analyzed on an intention-to-treat (ITT) basis in accordance with the study design. The Shapiro–Wilk test was used to assess data normality. All data analysis was performed using IBM® SPSS Statistics version 27.0 (IBM Corp, 2020). A *p*-value of <0.05 was considered significant.

### 3.8 Ethical considerations

Ethical clearance has been obtained from the Ethical Committee on the Use of Human and Animal Subjects in Teaching and Research of the College (reference no. REC2020076). The study was performed in accordance with the Declaration of Helsinki and relevant institutional guidelines and regulations. The study procedure was explained to the participants with a written study information sheet. Written informed consent was obtained from the participants prior to the commencement of the study. The participants were fully informed in writing about the purpose of the study. They were allowed to withdraw from the study without any consequences. The trial was registered with the research registry (Trial registration number: researchregistry8209).

## 4 Results

The commencement of the trial was postponed as a consequence of the COVID-19 outbreak, resulting in a delay in participant recruitment. Between July 2021 and September 2022, a total of 64 participants agreed to partake in the study and were randomly assigned to either the experimental or control groups. At T1, the experimental group had a retention rate of 71.9% (23 out of 32 participants), which decreased to 65.6% (21 out of 32). In the control group, the retention rate was 75% (24 out of 32) at T1 and 43.8% (14 out of 32) at T2. The experimental group (EG; *n* = 32) consisted of 22 females (68.8%) and 10 males (31.2%), with a mean age of 21.5 years (SD = 2.2). The control group (CG; *n* = 32) consisted of 25 females (78.1%) and 7 males (21.9%), with a mean age of 21.3 years (SD = 3.0). No significant differences between the groups except for religious belief. Table 1 displays the demographic characteristics of both groups.

**Table 1:** Characteristics of the participants.

	Total (*n* = 64)	Experimental (*n* = 32)	Control (*n* = 32)	Chi-square/*t*-tests *p*-value
Gender number (%)				
Female	47 (73.4)	22 (68.8)	25 (78.1)	0.396
Male	17 (26.6)	10 (31.2)	7 (21.9)	
Age (mean, SD)	21.4 (2.6)	21.5 (2.2)	21.3 (3.0)	0.811
Marital status				
Single	63 (98.4)	32 (100)	31 (96.9)	0.313
Married	1 (1.6)	0 (0)	1 (3.1)	
Religion				
Christian	11 (17.2)	1 (3.1)	10 (31.3)	0.011
Buddhism	4 (6.3)	2 (6.3)	2 (6.3)	
Worship	4 (6.3)	1 (3.1)	3 (9.4)	
None	45 (70.2)	28 (87.5)	17 (53.0)	
Living with				
Spouse	1 (1.6)	0 (0)	1 (3.1)	0.254
Parents	60 (93.8)	29 (90.6)	31 (96.9)	
Friends	1 (1.6)	1 (3.1)	0 (0)	
others	2 (3.0)	2 (6.3)	0 (0)	

Stress and anxiety are critical factors that can impact the well-being of undergraduate students. Therefore, the levels of stress and anxiety were categorized into different levels and presented in Table 2. The findings indicated that a majority (76.6%) of undergraduate students experienced moderate to high levels of stress, while approximately 60% reported mild to severe anxiety.

**Table 2:** Anxiety and stress levels of the participants (*N* = 64).

C-GAD-7	None/normal (0–4)	Mild (5–9)	Moderate (10–14)	Severe (15–21)
	Number (%)			
	24 (37.5)	25 (39.1)	11 (17.2)	4 (6.3)
C-PSS-10	Low (0–13)	Moderate (14–26)	High (27–40)	
	Number (%)			
	15 (23.4)	46 (71.9)	3 (4.7)	

C-PSS-10, Perceived stress; C-GAD-7, Generalized anxiety.

The outcome measures for both groups at three time points are presented in Table 3. It is evident that the mean scores of C-SOC-13, C-SCS-SF, and C-SMM exhibited an increase from T0 to T2, while there was a corresponding reduction in C-PSS-12 and C-GAD-7 scores in both groups. These findings indicate an improvement in these outcomes for both groups. Significant differences were found in the C-PSS-12 scores (*p* = 0.034) and C-GAD-7 scores (*p* = 0.009) at baseline. Independent t-tests revealed significant differences between the groups in C-SOC-13 scores (*p* = 0.021) and C-PSS-12 scores at week 10 (*p* = 0.046), as well as C-GAD-7 scores at week 6 (*p* = 0.022). However, when examining Table 4, he GEE analyses did not reveal any group effects or interaction effects (group x time) in C-SOC-13, C-SCS-SF, and C-SMM scores. Significant time effects were observed from C-SOC-13 scores (*p* = 0.007) at T2, as well as C-SCS-SF scores (*p* < 0.001) at T1 and T2. This indicates that the experimental group exhibited significant improvement in these outcomes over time, despite the absence of significant group or interaction effects. Furthermore, the results indicated that the time effect (Beta = −0.297, *p* = 0.000) and group x time effect (Beta = 0.245, *p* = 0.025) of C-PSS-12 were significant at T1. This suggests that the control group demonstrated a more significant decrease in stress levels compared to the experimental group by 0.245, indicating that GFMI may not be effective in reducing stress. Similar findings were observed for the C-GAD-7 scores, where the time effect (Beta = −0.344, *p* = 0.005) and group x time effect (Beta = 0.305, *p* = 0.045) of C-GAD-7 were significant at T2. This implies that the control group exhibited a more substantial decrease in anxiety levels compared to the experimental group by 0.305, suggesting that GFMI may not effectively reduce anxiety.

**Table 3:** Differences in outcomes between groups at baseline, week 6, and week 10.

Outcomes/Time	*n*	Experimental group		*n*	Control group		95% CI		*t*-tests *p*-value
		Mean (SD)					Lower Upper		
C-SOC-13									
Baseline	32	4.075	(0.763)	32	3.988	(0.673)	−0.446	0.273	0.632
Week 6	23	4.375	(0.866)	24	4.128	(0.613)	−0.691	0.198	0.269
Week 10	21	4.678	(0.876)	14	4.137	(0.416)	−0.992	−0.089	0.021
C-SCS-SF									
Baseline	32	3.166	(0.541)	32	3.061	(0.534)	−0.373	0.164	0.439
Week 6	23	3.457	(0.496)	24	3.445	(0.402)	−0.276	0.253	0.930
Week 10	21	3.511	(0.551)	14	3.352	(0.389)	−0.483	0.164	0.356
C-SMM									
Baseline	32	2.215	(0.534)	32	2.137	(0.433)	−0.321	0.165	0.523
Week 6	23	2.310	(0.484)	24	2.083	(0.535)	−0.527	0.074	0.136
Week 10	21	2.268	(0.583)	14	2.259	(0.417)	0.181	−0.377	0.961
C-PSS-10									
Baseline	32	1.678	(0.467)	32	1.972	(0.605)	0.023	0.564	0.034
Week 6	23	1.617	(0.601)	24	1.683	(0.506)	−0.260	0.392	0.686
Week 10	21	1.438	(0.422)	14	1.771	(0.528)	0.005	0.661	0.046
C-GAD-7									
Baseline	32	0.670	(0.566)	32	1.125	(0.765)	−0.951	−0.124	0.009
Week 6	23	0.584	(0.575)	24	0.988	(0.596)	0.060	0.748	0.022
Week 10	21	0.592	(0.474)	14	0.878	(0.444)	−0.039	0.611	0.083

C-SCS-SF, self-compassion; C-SOC-13, sense of coherence; C-SMM, stress-is-enhancing mindset; C-PSS-12, perceived stress; C-GAD-7, generalized anxiety.

**Table 4:** Treatment effects on all outcomes (GEE analyses).

	Experimental group		Control group		Time effect		Group effect		Time × group effect	
	Mean (SD)				Beta (SE)	*p*	Beta (SE)	*p*	Beta (SE)	*p*
C-SOC-13							−0.167 (0.165)	0.312		
T0	4.075	(0.763)	3.988	(0.673)	-	-	-	-	-	-
T1	4.375	(0.866)	4.128	(0.613)	0.170 (0.104)	0.101	-	-	0.094 (0.154)	0.541
T2	4.678	(0.876)	4.137	(0.416)	0.332 (0.123)	0.007	-	-	0.197 (0.193)	0.308
C-SCS-SF							−0.047 (0.122)	0.698		
T0	3.166	(0.541)	3.061	(0.534)	-	-	-	-	-	-
T1	3.457	(0.496)	3.445	(0.402)	0.365 (0.067)	0.000	-	-	−0.082 (0.118)	0.486
T2	3.511	(0.551)	3.352	(0.389)	0.344 (0.089)	0.000	-	-	−0.021 (0.140)	0.881
C-SMM							−0.104 (0.117)	0.377		
T0	2.215	(0.534)	2.137	(0.433)	-	-	-	-	-	-
T1	2.310	(0.484)	2.083	(0.535)	−0.026 (0.065)	0.693	-	-	0.088 (0.098)	0.366
T2	2.268	(0.583)	2.259	(0.417)	0.125 (0.077)	0.105	-	-	−0.073 (0.111)	0.507
C-PSS-12							−0.027 (0.102)	0.790		
T0	1.678	(0.467)	1.972	(0.605)	-	-	-	-	-	-
T1	1.617	(0.601)	1.683	(0.506)	−0.297 (0.077)	0.000	-	-	0.245 (0.110)	0.025
T2	1.438	(0.422)	1.771	(0.528)	−0.313 (0.104)	0.003	-	-	0.119 (0.1317)	0.367
C-GAD-7							−0.149 (0.104)	0.151		
T0	0.670	(0.566)	1.125	(0.765)	-	-	-	-	-	-
T1	0.584	(0.575)	0.988	(0.596)	−0.186 (0.111)	0.094	-	-	0.157 (0.136)	0.248
T2	0.592	(0.474)	0.878	(0.444)	−0.344 (0.123)	0.005	-	-	0.305 (0.152)	0.045

SE = standard error; C-SCS-SF, self-compression; C-SOC-13, sense of coherence; C-SMM, stress-is-enhancing mindset; C-PSS-12, perceived stress; C-GAD-7, generalized anxiety disorder; T0, baseline; T1, week 6; T2, week 10.

## 5 Discussion

Although stress exists as a normal part of life, constantly reacting to stressful situations without effective coping jeopardizes a person’s health and well-being (Chang et al., 2006). Over the long term, people who do not deal effectively with chronic stress are more likely to develop mental disorders and/or depression (Karyotaki et al., 2020). To reduce the physical, psychological, social, economic, and academic stress in college students, effective interventions are urgently needed. Our pilot study on the effects of the GFMI on undergraduate students yielded important insights. First and foremost, the majority of undergraduate students in our study experienced stress and anxiety, which is consistent with the findings of previous studies (Gardani et al., 2022) and highlights the need for interventions such as psychotherapy to alleviate their stress. However, our pilot study did not reveal any significant effects of GFMI in fostering coping resources to manage stress. These findings are consistent with previous studies that have also reported no significant change in sense of coherence (Brooks et al., 2010) and self-compassion (Sorensen et al., 2019) when using music as an intervention. It is important to note that sense of coherence and self-compassion are psychological constructs that may take time to develop (Kähönen et al., 2012; Lander, 2019). Limited studies have been conducted on the use of music interventions specifically targeting stress mindset. Previous studies aimed at enhancing stress mindset have incorporated background music as an adjunct to the intervention (Bosman, 2019; Girnth, 2020). However, the effects of music on stress mindset in these studies were inconclusive. Developing an enhanced mindset begins with self-awareness, which involves reflecting on one’s beliefs about intelligence, abilities, and personal qualities.

This pilot study only assessed outcomes over a duration of 10 weeks. A longer follow-up period, ranging from 6 to 12 months, would be necessary to determine whether the benefits of the GFMI are sustained over time. Additionally, the small sample size of our study may have contributed to the lack of statistical significance observed for various outcomes. Therefore, we recommend that future research endeavors include a larger sample size to enhance the statistical power and improve the reliability of the findings.

The GFMI did not demonstrate any effect in reducing stress and anxiety levels among the participants. In fact, the control group displayed a greater reduction in stress and anxiety. This could be attributed to the nature of the active control condition, which involved a relaxation program that also incorporated music listening, known for its stress-reducing effects (De Witte et al., 2020). Therefore, we suggest that a waitlist or no treatment control group be included in a full-scale efficacy trial.

This pilot study, albeit limited by a small sample size and lack of statistical power to detect effectiveness between groups, presented an invaluable opportunity to assess the feasibility and acceptability of the study procedures. Despite being conducted amidst the challenging circumstances of the COVID-19 pandemic, our study has yielded compelling evidence supporting the feasibility of the intervention, with an impressive recruitment rate of 82.1%. Furthermore, we have observed commendable retention rates in both the experimental group (71.9%) and the control group (75%) over the 6-week study intervention period, indicating active participation. However, some participants were lost to follow-up by week 10 due to their academic commitment. Moving forward, it is imperative to further refine the GFMI and ensure that it is adequately supported by resources and that a well-defined protocol is in place. It is essential to incorporate flexibility in the schedule to encourage high response rates in completing the follow-up data.

### 5.1 Strengths and limitations

This trial has several major strengths. Firstly, it is among the pioneering studies to investigate the effects of GFMI in stress management and may be the first study to utilize this therapy specifically for college students. The components of GFMI are aligned with the cognitive and emotional aspects of coping resources, which offers a high potential for targeting the key mechanisms of stress management. Secondly, the group-based control condition includes components that are similar to GFMI, such as guided imagery, music listening, and drawing, enabling a direct comparison of the specific intervention mechanism of GFMI. Thirdly, music listening is a popular leisure activity among young people (Miranda, 2019), making it more likely that young individuals will accept this type of intervention and participate in the study. However, considering the heavy study load of the participants, we recommend a more flexible schedule to increase compliance and maximize the intervention’s potential benefits.

Our study has identified several limitations. Firstly, music therapists cannot be blinded, which may introduce bias in the delivery of the intervention. Secondly, the outcomes are measured with self-reported questionnaires, which may be compromised with social desirability bias. In addition to self-reported questionnaires, future studies could use biological markers of stress (e.g., cortisol levels) to minimize bias. Thirdly, we acknowledge that certain confounding variables, such as participants’ music education or professional background, and hormonal fluctuations related to the menstrual cycle, have been shown to impact mood, emotional processing, and physiological reactivity in females (Protopopescu et al., 2008; Ossewaarde et al., 2010). In this pilot study, we did not measure or account for the menstrual cycle phase of female participants, which could limit the interpretation of our findings. Therefore, it is recommended that future studies include measures of participants’ music education and music professional activity as part of the demographic information. Lastly, it should be cautious to interpret the results due to the relatively high attrition rate observed in the control group at T2.

## 6 Conclusion

As a pilot study, our research offers preliminary evidence supporting the feasibility of implementing the GFMI for young adults. The findings derived from our study will serve as a foundation for conducting a more extensive investigation to ascertain the effectiveness of the music imagery intervention in enhancing coping resources for stress management among young adults. In this pilot phase, it is noteworthy that the GFMI did not yield statistically significant effects in promoting coping resources to reduce stress and anxiety. To establish the reliability and validity of the GFMI in providing coping resources for stress and anxiety management within this population, future studies with larger sample sizes are warranted.

## Data availability statement

The raw data supporting the conclusions of this article will be made available by the authors, without undue reservation.

## Ethics statement

The studies involving humans were approved by The Ethical Committee on the Use of Human and Animal Subjects in Teaching and Research from Tung Wah College, Hong Kong (reference no. REC2020076). The studies were conducted in accordance with the local legislation and institutional requirements. The participants provided their written informed consent to participate in this study.

## Author contributions

WC: Conceptualization, Data curation, Formal Analysis, Funding acquisition, Investigation, Methodology, Project administration, Supervision, Writing – original draft, Writing – review and editing. LW: Data curation, Investigation, Project administration, Writing – review and editing.
